# Integrative taxonomy: Combining morphological, molecular and chemical data for species delineation in the parthenogenetic *Trhypochthonius tectorum *complex (Acari, Oribatida, Trhypochthoniidae)

**DOI:** 10.1186/1742-9994-8-2

**Published:** 2011-02-08

**Authors:** Michael Heethoff, Michael Laumann, Gerd Weigmann, Günther Raspotnig

**Affiliations:** 1 Institute of Zoology, Karl-Franzens University Graz, Universitätsplatz 2, 8010 Graz, Austria; 2 Institute for Evolution and Ecology, University Tübingen, Auf der Morgenstelle 28E, 72076 Tübingen, Germany; 3 Insitute of Zoology, Free University Berlin, Koenigin-Luise-Straße 1-3, 14195 Berlin, Germany

## Abstract

**Background:**

There is a long-standing controversial about how parthenogenetic species can be defined in absence of a generally accepted species concept for this reproductive mode. An integrative approach was suggested, combining molecular and morphological data to identify distinct monophyletic entities. Using this approach, speciation of parthenogenetic lineages was recently demonstrated for groups of bdelloid rotifers and oribatid mites. *Trhypochthonius tectorum*, an oribatid mite from the entirely parthenogenetic desmonomatan family Trhypochthoniidae, is traditionally treated as a single species in Central Europe. However, two new morphological lineages were recently proposed for some Austrian populations of *T. tectorum*, and were described as novel subspecies (*T. silvestris europaeus*) or form (*T. japonicus *forma *occidentalis*). We used the morphological and morphometrical data which led to this separation, and added mitochondrial and nuclear DNA sequences and the chemical composition of complex exocrine oil gland secretions to test this taxonomical hypothesis. This is the first attempt to combine these three types of data for integrative taxonomical investigations of oribatid mites.

**Results:**

We show that the previous European species *T. tectorum *represents a species complex consisting of three distinct lineages in Austria (*T.tectorum*, *T. silvestris europaeus *and *T. japonicus *forma *occidentalis*), each clearly separated by morphology, oil gland secretion profiles and mitochondrial *cox1 *sequences. This diversification happened in the last ten million years. In contrast to these results, no variation among the lineages was found in the nuclear 18S rDNA.

**Conclusions:**

Our approach combined morphological, molecular and chemical data to investigate diversity and species delineation in a parthenogenetic oribatid mite species complex. To date, hypotheses of a general oribatid mite phylogeny are manifold, and mostly based on single-method approaches. Probably, the integrative approach proposed here can be used to uncover further hidden biodiversity of glandulate Oribatida and help to build up more stable phylogenetic hypotheses in the future.

## Background

More than twenty hypotheses try to explain the advantages of sexual reproduction over parthenogenesis or asexuality [1,2]. Most of these theories tolerate the existence of parthenogenetic species in the short-term, but predict that there should be no radiation and long-term survival of groups lacking sexual reproduction. About 2,000 parthenogenetic species have been described among almost all groups of animals [3]. However, existence and recognition of parthenogenetic species remains a controversial topic, mostly due to the fact that the traditional biological species concept is axiomatically related to sexuality. Additionally, misunderstandings of parthenogenetic population genetics have led to the prediction that parthenogenetic organisms must form a continuum of genetic variation [4]. But this is not necessarily true - parthenogenetic lineages can split into independently evolving entities, thus speciation of parthenogens can be addressed empirically [5]. Recently, speciation of ancient parthenogenetic lineages has been demonstrated for bdelloid rotifers [4,6-8] and several groups of oribatid mites [9-13]. High and consistent clonal diversity was also demonstrated for the putative ancient parthenogenetic *Darwinula stevensoni *(Ostracoda) [14], contrasting the low diversity shown earlier [15].

The existence of parthenogenetic species has been proposed in different species concepts, including the evolutionary, ecological and phylogenetic species concepts [16-18], but it remains a major concern how a parthenogenetic species can be defined in a biological meaningful context. Recently, a new evolutionary genetic species concept, based on population genetic theory and DNA sequence data, has been proposed and applied to delineate parthenogenetic species of bdelloid rotifers and oribatid mites [19,20]. Another DNA-sequence based approach, genetic barcoding, uses a part of the mitochondrial cytochrome oxidase 1 (*cox1*) gene to differentiate between species on the basis of genetic distances and was proposed to be useful for the identification of undescribed species [21-23]. However, this pure molecular-based barcoding was criticized [24-27] to be a phenetic, non-cladistic approach and no general definition is available for the amount of genetic distance indicating a separation of lineages into species. Hence, an integrative approach was suggested, combining data from multiple sources for the identification and definition of new species [28-31] and such integrative approaches using molecular and morphological data were successfully used for the identification of independently evolving lineages within parthenogenetic clusters of bdelloid rotifers [6] and the parthenogenetic oribatid mite genus *Tectocepheus *[13]. However, it was suggested that at least three different sources of data should be included for a reliable delimitation of species boundaries [30,31]. Besides morphological and molecular data, we included the chemical composition of oil gland secretions to investigate characteristics of Austrian populations of the oribatid mite *Trhypochthonius tectorum*.

Oil glands are paired opisthosomal sac-like exocrine glands characteristic of the so-called 'glandulate Oribatida' [32] and may contain complex mixtures of terpenes, aromatics, hydrocarbons [33] and alkaloids [34]. The chemical composition of oil gland secretions was shown to be a phylogenetically informative set of characters [35], allowing also differentiation between populations of parthenogenetic oribatid mite species [33].

Oribatid mites are a speciose group of chelicerates (~10.000 species, [36]) with Devonian [37], Silurian [38] or Precambrian [39] origin. Parthenogenesis is widespread among the Oribatida and several large monophyletic and parthenogenetic groups exist, consisting of 50 to 180 morphologically described species [10,12]. One of these exclusively parthenogenetic families, the Trhypochthoniidae [40], comprises 51 species [41] with about 25 species in the genus *Trhypochthonius *[42]. Parthenogenetic reproduction of Trhypochthoniidae was first assumed by Grandjean in 1941, based on the rarity of males [43], and later experimentally proven for numerous species of this family [44-46]. *Trhypochthonius tectorum *[47] was reported from Holarctic, Oriental and Neotropic regions and a number of subspecies have been described using morphology only [41], although their identity is questionable [40]. Previously assumed as a single species, *Trhypochthonius tectorum *was recently hypothesized to be a species complex rather than a single species in Austria, and a new subspecies (*T. silvestris europaeus*) as well as a new form (*T. japonicus *forma *occidentalis*) have been differentiated from *T. tectorum *s. str. using morphological data [48]. Here, we expand this morphological analysis of Austrian populations by including molecular and chemical data to test the hypothesis of independent entities using an integrative approach.

We show that the three lineages proposed by [48] are independent entities, clearly separated by morphology, gland secretions and mitochondrial sequences and that completely homogeneous nuclear ribosomal DNA contrasts this separation.

## Results

### Chemical analyses

**Figure 1:**
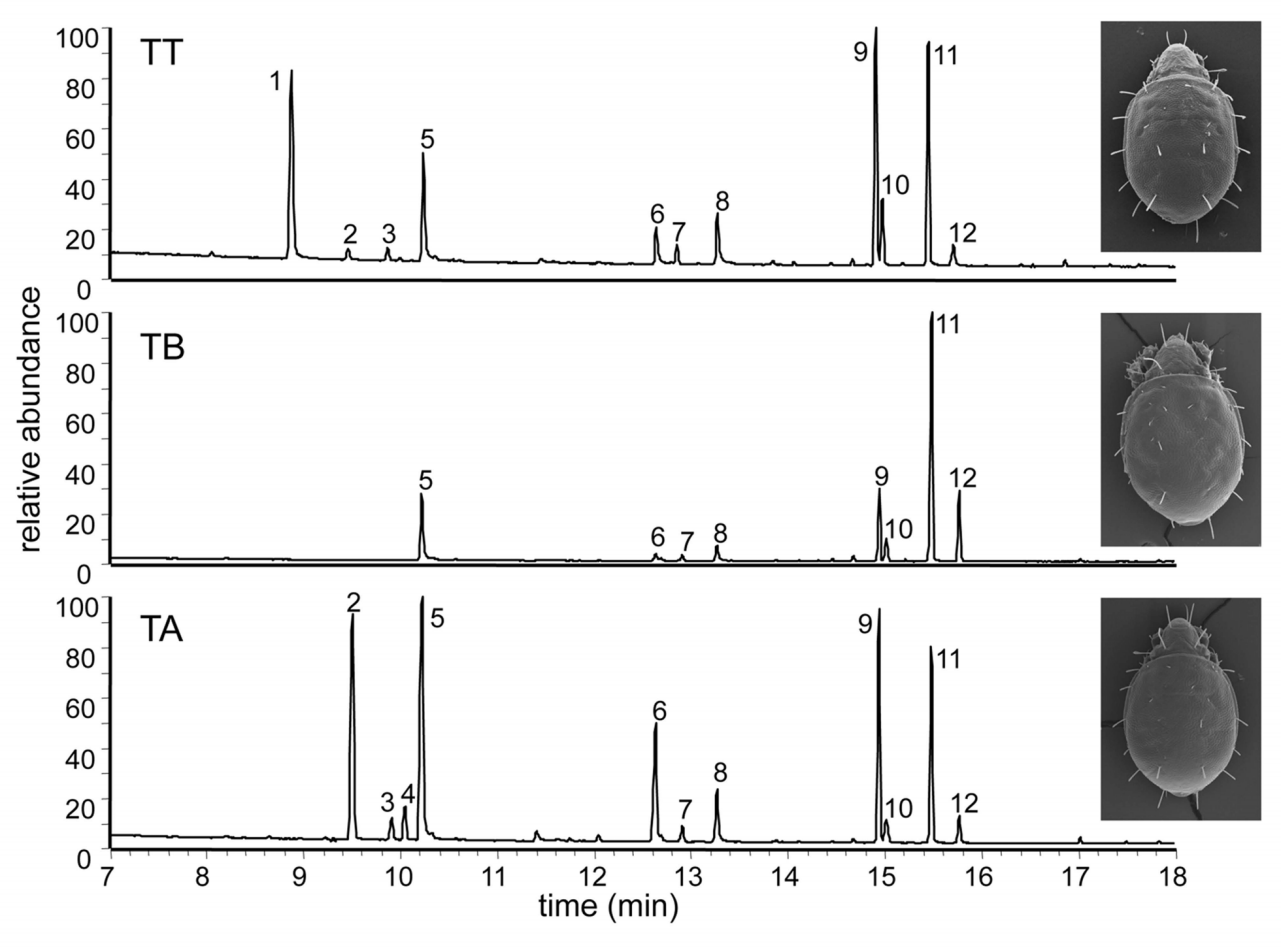
**Chemical profiles**. Chromatographic profiles of extracts from TT, TA and TB.

Analyses of oil gland secretion profiles led to three distinct chemical profiles (Figure 1, Table 1). One of the gas chromatographic profiles was identical to published data of *T. tectorum *[49] hence the lineage showing this profile was denoted as *T. tectorum *(TT) for morphometrical and molecular analyses. The chemical profile of TT consisted of eleven compounds with characteristic relative abundance (Table 1). The compounds were 2-hydroxy-6-methylbenzaldehyde (= 2,6-HMBD; peak 1), neral (peak 2), geranial (peak 3), 2-formyl-3-hydroxy benzaldehyde (= 2,3-FHBD, = γ-acaridial; peak 5), pentadecane (peak 7), 6,9-heptadecadiene (peak 9, identified by DMDS-derivatives), heptadecene (peak 10, double bond position not identified, probably 4-heptadecane), (Z,E)-farnesal (peak 11), (E,E)-farnesal (peak 12) and two unknown components (peaks 6, 8). The described profile was consistently found in all extracts of TT from any location (CF, SG and SB; see Methods for locations). In contrast to this already well-known profile of *T. tectorum*, the profiles of *T. silvestris europaeus *(denoted as TA) and *T. japonicus *forma *occidentalis *(denoted as TB) were considerably different. The TA profile from collection site CW (see Methods for location), lacked 2,6-HMBD, but in addition showed small amounts of neryl formate (Figure 1, peak 4). TB was syntopically found at sample site CW and the chemical profile lacked 2,6-HMBD, neral, geranial and neryl formate. Hence, the TB-profile consisted of eight compounds only (Figure 1). An outgroup comparison was done with *Archegozetes longisetosus*, confirming the already published ten compounds-profile of 2,6-HMBD, neral, geranial, neryl formate, γ-acaridial, pentadecene, n-pentadecane, heptadecadiene, heptadecene and, although only in trace quantities, heptadecane [50,51].

**Table 1:** Oil gland secretion profiles

	TT	TA	TB	AL
2,6-HMBD	**28.4 ± 5.9**	0	0	4.4 ± 1.9
Neral	0.8 ± 0.3	**21.4 ± 3.1**	0	**16.1 ± 2.7**
Geranial	0.9 ± 0.5	1.8 ± 0.6	0	0.6 ± 0.2
Neryl formate	0	1.5 ± 1.2	0	**34.8 ± 4.4**
γ-Acaridial	**12.8 ± 3.1**	**29.2 ± 8.3**	**15.5 ± 4.4**	**25.5 ± 4.9**
Unknown_1	3.6 ± 1.2	8.7 ± 2.6	3.1 ± 2.1	0
Pentadecene	0	0	0	2 ± 0.6
Pentadecane	1.4 ± 0.4	1.3 ± 0.3	1.2 ± 0.3	**10.4 ± 2.3**
Unknown_2	4.4 ± 1.6	3.4 ± 1.5	6.6 ± 4.9	0
Heptadecadiene	**21.6 ± 5.0**	**14.2 ± 2.7**	**12.5 ± 2.2**	0.4 ± 0.2
Heptadecene	4.7 ± 1.6	2.2 ± 0.5	4.7 ± 0.6	5.7 ± 2.4
Heptadecane	0	0	0	0.1 ± 0.1
Z,E-Farnesal	**19.4 ± 4.1**	**14.0 ± 5.2**	**45.6 ± 7.5**	0
E,E-Farnesal	2.0 ± 1.0	2.3 ± 1.2	**10.6 ± 3.4**	0

Composition of oil gland secretions (TT, n = 14; TA, n = 13; TB, n = 4), *Archegozetes longisetosus *(AL, n = 33, see also [51]); values represent % of whole secretion (average ± SD); main components (>10%) in bold.

**Figure 2:**
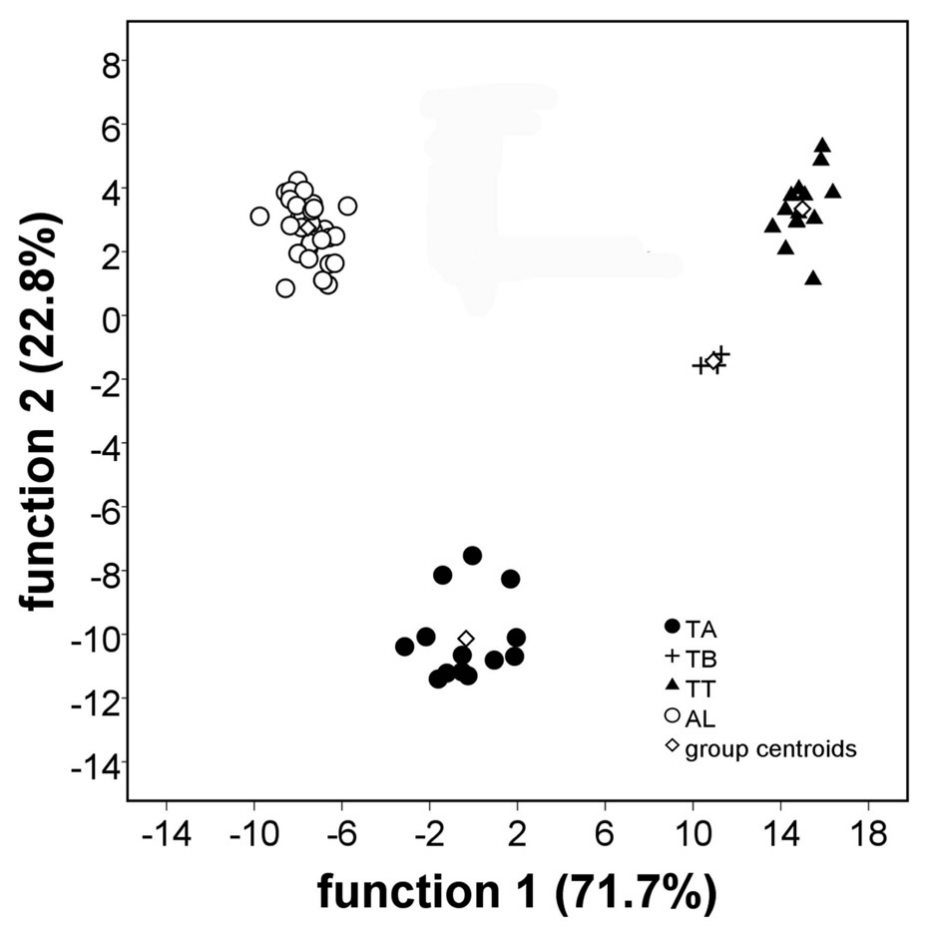
**Canonical discriminant analysis of oil gland secretions**. Estimation of the validity of the discriminant function is based on the significance of Wilk's Lambda and the percentage of correct assignment. Four chemical groups were verified, clearly indicating that TT, TA, TB and AL are chemically completely separated (100% of cases were correctly assigned to the four previously defined taxa).

Apart from easily visible qualitative differences, all profiles were quantified (leading to characteristic patterns of relative abundance of components in each profile (Table 1), and were subsequently subjected to multivariate statistics, forming consistent and significant clusters that do not overlap (Figure 2).

### Morphometrical analyses

**Figure 3:**
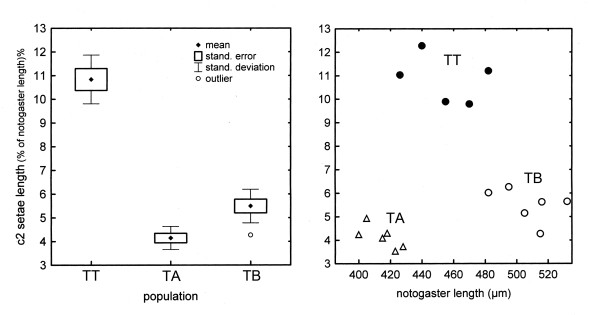
**Lengths of setae**. Ranges of length-values of notogaster setae *c*_2 _(**left**) and individual length-values plotted against notogaster lengths (**right**) of populations TT, TA and TB from Austria.

**Figure 4:**
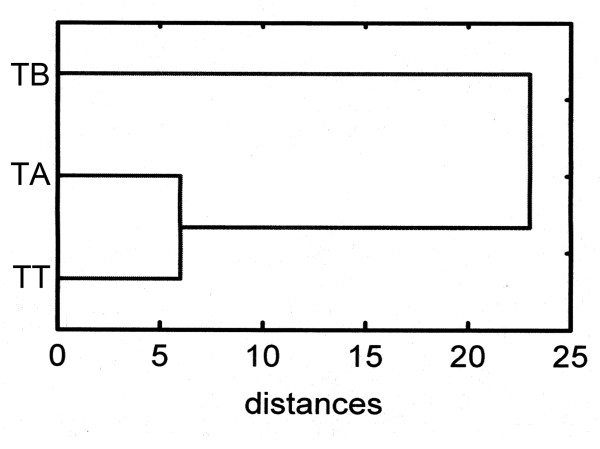
**Morphometric similarities**. Tree cluster on the similarities among TT, TA and TB. Distances calculated from summed differences of notogastral setae types (see [48]).

Details of morphometrical measurements are given in [48]. Here, we only shortly summarize the main results that we used within this integrative framework. TT, TA and TB differed significantly in body length. With a mean body length of 643 μm, TT was larger than TA (mean: 597 μm), but smaller than TB (mean: 717 μm). Besides this, TT, TA and TB could be separated by their different numbers of genital setae and their relative length of the notogastral setae *c*_*2*_*, d*_*1*_*, d*_*3*_*, e*_*1 *_and *p*_*3 *_(exemplified for *c*_*2 *_in Figure 3). In addition, distance-based cluster analyses of the setae types show a clear separation of the three groups with a higher similarity of TT and TA than any of these has to TB (Figure 4).

### Molecular analyses

**Figure 5:**
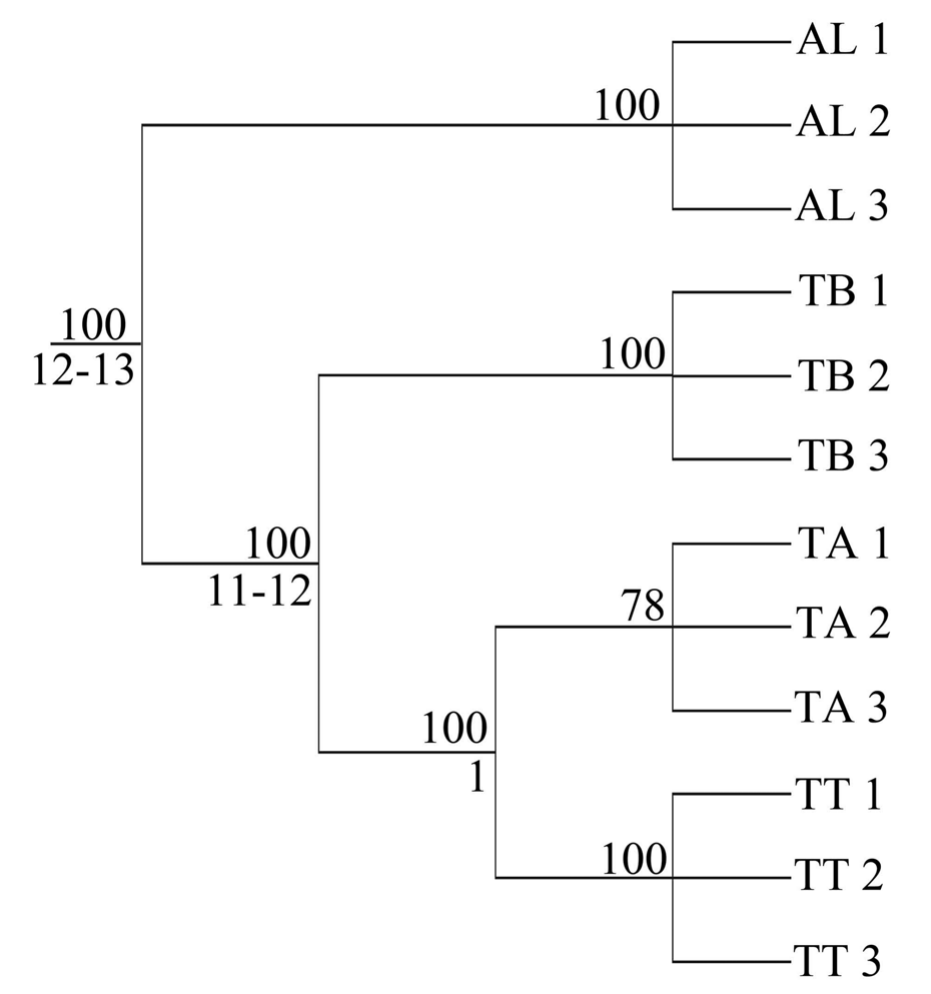
**Phylogenetic analysis**. Maximum parsimony cladogram of TT, TA, TB with the outgroup AL. Tree length: 218, CI: 0.9954, RC: 0.9937. Numbers on branches are bootstrap values (1,000 replicates). Numbers below branches indicate the estimated age of the split (myr).

A 600 bp fragment of the *cox1 *gene was obtained from each three specimens of TT, TA and TB and the outgroup AL and aligned by hand without any ambiguity or gaps. No variation was found within the replicates of TT, TA, TB and AL. In total, 181 (30.2%) nucleotide positions were variable and informative. Excluding the outgroup, 127 bp (21.2%) were variable and informative among the three *Trhypochthonius *groups TT, TA and TB. TT was characterized by eleven apomorphic nucleotide positions, TA showed two apomorphies, and for TB there were 73 apomorphic characters. TT and TA showed 82 synapomorphies, contradicted by three positions shared by TA and TB. Not a single synapomorphy was found for TT and TB. Maximum Parsimony analyses in PAUP* resulted in a single tree with a tree-length of 218 and consistency index (CI) and rescaled consistency index (RC) of 0.99 each (Figure 5). The identical topology was found with Maximum Likelihood analyses.

Relative rate tests using AL as outgroup and all combinations of TT, TA and TB as ingroup taxa resulted in no significant rate variations (χ²(1) < 3.6, p > 0.05). In addition, a likelihood ratio test, based on the likelihoods of the corresponding branch-and-bound trees, was performed with the molecular clock enforced and not enforced (enforced: -ln L = 1679.427; not enforced: -ln L = 1674.96; χ²(10) = 0.104, p > 0.99) and showed also no rate variation. Therefore, the assumption of a molecular clock seems appropriate. A molecular divergence rate of 2.15% per million years was estimated for the *cox1 *gene of oribatid mites [12,52]. Genetic pairwise p-distances were corrected by an evolutionary model (HKY, [53]; estimated by hLRTs, AIC and BIC in Modeltest 3.7, [54]) with nucleotide composition A: 0.2531, C: 0.2198, G: 0.19, T: 0.3371 and k: 1.5108 (Table 2). Evolutionary ages of the lineages were estimated based on the corrected distances: TT and TA separated about one million years ago and the last common ancestor of TB and TT/TA lived about eleven million years ago (Figure 5).

**Table 2:** Genetic distances

Split	p-distance	HKY distance	Age of split [myr]
AL - TT	0.23167	0.28009	13
AL - TA	0.22167	0.26438	12
AL - TB	0.21667	0.25776	12
TT - TA	0.02167	0.022	1
TT - TB	0.21	0.2498	12
TA - TB	0.195	0.22863	11

Genetic pairwise p-distances and corrected distances (HKY) with estimated ages of lineages (based on corrected distances).

The alignment of the nuclear 18S rDNA consisted of 1700 nucleotides. All *Trhypochthonius*-sequences were identical, and all were identical to a published sequence of *T. americanus *(EF081298, [11]). Hence, no further phylogenetic analyses of these sequences were performed.

## Discussion

What is a parthenogenetic species? We do not aim to give an exhaustive discussion on this difficult topic, but we want to shade light on some practical implications, i.e. methods to detect separated genotypic and phenotypic entities (whether they will be denoted as species or not). It is clear that reproductive isolation, the basis of the biological species concept [55], is meaningless for the definition of parthenogenetic species. Almost all parthenogens are described on their morphology only (morphospecies), applying at best the same subjective criteria for discriminating species as taxonomists do with sexual species. The occurrence of phenotypic plasticity or the absence of phenotypic variation despite genotypic variability ('cryptic species', see [12]) can be observed in many groups irrespective their mode of reproduction. Therefore, both may be analyzed with the same procedures. However, the delineation of species and their subsequent classification into larger taxonomic units may be somehow problematic, especially when dealing with character-poor organisms of small size and similar morphology. Many soil-dwelling arthropods belong to this group, such as some highly-conservative opilionids of different suborders [56,57], but also many Oribatida. In these groups numerous so-called 'species-complexes' exist, i.e. assemblages of similar species or sub-species that are not clearly delineated from each other. Such 'species' may either show a high intraspecific variability of characters or may actually represent groups of closely related, cryptic (or nearly cryptic) species. In many cases, one set of characters alone - e.g. traditional characters from external morphology - fails to answer questions on this low taxonomic level. Hence, numerous approaches towards integrative taxonomy have been attempted in the last years: using a combination of methods, a more rigorous concept of the delimitation of problematic species has been introduced [30,31]. Many examples of the successful application of combined methods meanwhile exist, and with respect to the Opiliones mentioned above, a large number of new but so far cryptic opilionid *Cyphophthalmus*-species on the Balkan Peninsula have been discovered using morphological and molecular characters [57,58]. By contrast, the systematics of Oribatida suffers greatly from still uni-methodological approaches: i) the majority of taxonomic studies in the Oribatida is still exclusively based on traditional sets of data derived from external morphology; and ii) novel methods, such as molecular phylogenetic approaches, are rarely combined with morphological data. Molecular data for the delineation of the parthenogenetic oribatid mite genus *Tectocepheus *were presented [13] and combined with morphological data from [59] to demonstrate parthenogenetic radiation - a rare example of an integrative approach in oribatid mite systematics.

Each uni-methodological approach, including molecular techniques, is assumed to have an inherent failure rate in the delimination of species [31]. With respect to taxonomic studies in arthropods, and according to [31], the failure rate is 28% when using nuclear DNA-data alone, and 33% when using mitochondrial DNA. Failure rates arising from studies using morphological or chemical data alone show similar failure rates of 23% and 22%, respectively. Combining any two of these methods leads to a reduced failure rate of 9%, but only when three are combined, a statistically acceptable failure rate below 5% can be achieved [31].

### Chemical data

With respect to glandulate Oribatida and their multicomponent secretions from the oil glands, an independent pool of characters has been made available to oribatid systematics and phylogeny in the last years [33,35]. One model group for such studies is the Trhypochthoniidae, medium to large oribatids that i) possess largely developed oil glands, making it possible to analyze individual extracts in some species, ii) show specific combinations of chemically already characterized compounds (so-called 'Astigmata compounds' *sensu *[60]), and iii) generally exhibit information-rich multi-component secretion profiles. In addition, a considerable data base on their secretions has been generated, representing an important source for reference: in detail, secretion profiles of *Archegozetes longisetosus *[50,51], *Trhypochthoniellus crassus *and three species of *Trhypochthonius *(*T. tectorum*, *T. japonicus *and a not determined Japanese *Trhypochthonius *species) have already been analyzed, each showing species-specific and interspecifically distinctive secretion profiles [49,61,62]. Considering these data, the profile of TT appears to be rather basal within Trhypochthoniidae, showing the full spectrum of 'Astigmata compounds' except for neryl formate. The lack of 2,6-HMBD in TA and TB, however, may be due to convergent reduction, especially when regarding the clear phylogenetic relatedness of TT and TA implied by molecular data.

### Morphological data

In a morphometrical analysis that was the initiation of this integrative project (details in [48]) the three distinct European lineages within *Trhypochthonius tectorum *s. lat. (TT, TA, TB) were compared with *T. japonicus *[63] from Japan, *T. americanus *[64] and *T. silvestris *[65], both from North America. TA looked quite similar to *T. silvestris*, but was statistically distinct, and hence was proposed as subspecies *T. silvestris europaeus *[48]. TB was very similar to *T. japonicus*, the difference in morphometric respect was small, but partly significant, and thus TB was classified as geographically distinct *T. japonicus *forma *occidentalis *[48]. This close relationship is also supported by oil gland chemistry showing nearly identical secretion profiles of TB and *T. japonicus *[62]. Since there is a graduated degree of similarities with respect to the morphological characters within the lineages, these were expressed taxonomically as form or subspecies [48]. Morphologically, species of *Trhypochthonius *show several evolutionary lineages, one of these is the *T. tectorum *species complex [48], which - apart from *T. tectorum *- contains several other representatives from, e.g., North America and Japan.

### Molecular data

Phylogenetic analyses using the maximum parsimony criterion are prone to the phenomenon of long-branch attraction, especially when molecular data are used and divergences between sequences are high [66]. Hence, if long branches occur in the data, an alternative method, such as maximum likelihood, is desired. However, if no long branches exist in the data, the data-set is small enough (less than 25 taxa) to be analyzed exhaustively (i.e. with a guarantee to find the shortest tree), only a single shortest tree is to be found, and the distribution of characters is highly congruent on this shortest tree, then we see no good reason to use other methods than maximum parsimony (however, we performed also maximum likelihood analyses with identical results). In this study, the 600 bp *cox1*-alignment of the *Trhypochthonius*-species showed 127 variable and phylogenetically informative nucleotide positions (21.2%). There were clear apomorphies for each of the three lineages, and a high number (82) of synapomorphies for the sister-taxa TT and TA, contradicted by only three nucleotide positions supporting TA+TB. This results in a high consistency (and rescaled consistency) index of 0.99, very close to complete congruence (Figure 5). However, the bootstrap-support for the monophyly of TA is only 78, which can be explained by the low (but consistent) number of only two apomorphies that define this taxon. There is not a single position that supports any other hypotheses than the monophyly of TA, but the two apomorphies simply get lost by chance in 22% of the bootstrap resampling procedure. Hence, we think that the high amount of informative positions and the high consistency index clearly support the topology given in Figure 5.

In contrast to the high divergence and information of the *cox1*-sequences, the 1,700 bp alignment of the nuclear ribosomal 18S sequences showed no variation at all. We included a published sequence of *T. americanus *(EF081298) in the alignment, and this sequence also was identical. This phenomenon is not unique among mites - Navajas et al. [67] reported 5% of divergence in the mitochondrial COI sequences and no variability in the ribosomal nuclear ITS2 sequences in the spider mite *Tetranychus urticae*. This was explained by a high colonization potential of this species, preventing long-term differentiation. However, *T. urticae *is a sexually reproducing, thus recombining pest-species, and *T. tectorum *is a parthenogenetic species belonging to the Desmonomata, hence presumably has an inverted meiotic sequence and no meiotic recombination [68-71]. Another ancient parthenogenetic species, *Darwinula stevensoni *(Ostracoda) also showed this same pattern: homogenized nuclear ribosomal sequences in contrast to divergent mitochondrial COI sequences [15]. Here, this pattern was explained by a reduced mutation rate and effective machinery for DNA repair. We do not exactly know ultimate causes for the contrasting nuclear and mitochondrial divergence in *T. tectorum*, but we think that besides a lower mutation rate of the nuclear genome this could be a result of the special reproductive mechanism: automixis with inverted meiosis and terminal fusion [70].

The *Trhypochthonius tectorum *complex was hitherto conceived as a single species in Europe. Our integrative approach shows consistently that i) the recently proposed *T. silvestris europaeus *and *T. japonicus *forma *occidentalis *are distinct taxonomical entities, ii) *T. tectorum *and *T. silvestris europaeus *are related taxa which separated about one million years ago, iii) *T. japonicus *forma *occidentalis *separated from *T. tectorum *and *T. silvestris europaeus*11-12 million years ago.

## Conclusions

We showed that an integrative approach, combining morphometrical, chemical, and molecular data, could be used to identify distinct lineages within a parthenogenetic oribatid mite species complex. A combination of these three methods might also help in unraveling at least some of the numerous controversies in glandulate oribatid mite phylogeny.

The two new lineages *T. silvestris europaeus *and *T. japonicus *forma *occidentalis *were found by taking only a few, random samples in Austria. Hence, we assume that a more thorough sampling all over the Holarctic range of distribution will probably uncover numerous additional lineages within the *T. tectorum *complex. Thus, unless this complex is investigated in more detail, and to avoid further confusion, the recently proposed taxonomical rank of *T. silvestris europaeus *(subspecies) and *T. japonicus *forma *occidentalis *(form) is presently left unchanged.

A future agreement for the definition of parthenogenetic species in an integrative context seems desirable, especially since more and more different sources of data (morphological, molecular, chemical, biochemical, physiological, ecological, behavioural) are included in integrative approaches.

## Methods

### Specimens

Four sites in Austria were sampled; specimens of *T. tectorum *were collected by hand and kept alive for individual extraction and chemical analyses of oil gland secretion profiles. Subsequently, specimens were sorted with respect to their secretion profiles and size, stored in ethanol and analyzed morphometrically and genetically. Sample sites were: (1) Carinthia, Ferlach, moss on a roof (= CF); (2) Carinthia, Waidischbach, moss and litter in a *Pinus *stand (= CW); (3) Styria, Graz, moss on a street pavement (= SG); (4) Styria, Bachsdorf, moss on a roof (= SB).

The laboratory lineage *A. longisetosus *ran (= AL, [72]), also a member of the parthenogenetic Trhypochthoniidae, originated from our laboratory culture and was used as outgroup for phylogenetic analyses of molecular data and for comparisons of oil gland chemistry.

### Chemical analyses

Specimens were handled with care to avoid release of their oil gland secretions prior to extraction. Extracts were prepared by submersing living individuals in 50 μl of hexane for 30 minutes for a discharge of secretions into the solvent [73]. Crude extracts were used for chemical analyses using a Trace gas chromatograph (GC) coupled to a Voyager mass spectrometer (MS) (both from Thermo, Vienna, Austria). The GC-column (ZB-5MS fused silica capillary column: 30 m × 0.25 mm i.d., 0.25 μm film thickness; Phenomenex, Aschaffenburg, Germany) was directly connected to the ion source of the MS. The splitless Grob injector was kept at 260°C, and helium was used as a carrier gas with a constant flow rate of 1.5 ml/min. The temperature program was set to 50°C (1 min), followed by an increase of 10°C/min until 200°C were reached, then 15°C/min until 300°C were reached with a final isothermal hold (300°C) for 5 minutes. The ion source of the MS was kept at 150°C and the transfer line at 310°C. Electron impact spectra were recorded at 70 eV.

Where possible, compounds were identified on the basis of mass spectral data and comparison of retention times to authentic standards or tentatively, by interpretation and comparison of mass spectra to reference spectra from literature and the NIST-library [73].

Secretion profiles were evaluated by integration of peak areas in the chromatograms and by calculation of the relative abundance of peaks (given in % of peak area of the whole secretion). Secretion profiles, including qualitative and quantitative information, were further subjected to discriminant analyses (using SPSS 16). Compounds were treated as variables, and the profiles evaluated represented the 'cases' for analyses. Stepwise discriminant analyses were carried out to determine whether the previously (morphologically) defined groups (4 species) could be discriminated on basis of their chemical profiles and to evaluate which compounds mainly discriminated between groups. Wilk`s Lambda and the percentage of correct assignment were used to estimate validity of discrimination.

### Morphometrical analyses

Specimens were macerated in lactic acid and mounted in open cavity slides covered partly by a cover glass, which allows turning each specimen for microscopic analyses from all perspectives. Details of measurements are given in [48].

Each statistical analysis for setae and notogaster lengths was performed as Kruskal-Wallis-ANOVA-test (H-test) for multiple tests over all populations. In cases of significance, subsequently a pairwise Mann-Whitney-Median-test (U-test) was used for detecting the significant differences between population pairs.

The multi-dimensional cluster analysis of the qualitative differences of notogastral setae between the populations were based on setal types where each of the 15 setae (*c*_1 _- *p*_3_) represents one dimension. The pairwise numerical differences between the *Trhypochthonius *populations of all notogastral setal types were used for a tree-cluster analysis (complete linkage of all Manhattan-City-Block-distances).

### Molecular analyses

Total DNA was extracted from single specimens using the DNeasy Tissue Kit (Qiagen, Hilden, Germany) according to the manufacturer's protocol. PCR was performed with the HotStarTaq Master Mix kit (Qiagen, Hilden, Germany); the total reaction volume of 20 μl contained 1.5 mM MgCl_2_, 100 pmol of each primer, 200 μM of each dNTP and 1 Unit of Taq-polymerase.

A 600 bp fragment of the mitochondrial *cox1 *gene, corresponding to the amino acid positions 19-218 of the *Steganacarus magnus *(Oribatida) *cox1 *protein [74] was obtained with the primers and protocol given in [12]. Nuclear sequences of the 18S rDNA (1,700 bp) were amplified using primers and procedure described in [13]. Sequencing was performed in both directions on an ABI capillary sequencer. Sequences were deposited in GenBank (18S data set: *A. longisetosus *HQ661379, *T. silvestris europaeus *HQ661380-HQ661382, *T. japonicus *forma *occidentalis *HQ711366-HQ711368, *T. tectorum *HQ711369-HQ711371; *cox1 *data set: *A. longisetosus *HQ711372, *T. silvestris europaeus *HQ711373-HQ711375, *T. japonicus *forma *occidentalis *HQ711376-HQ711378, *T. tectorum *HQ711379-HQ711381).

Sequences were verified to be of oribatid mite origin by comparisons with known sequences in GenBank using the BLASTN search algorithm [75] and aligned by hand in BioEdit 7 [76]. Models for sequence evolution and corresponding parameters were estimated using hierarchical likelihood ratio tests (hlrts) with Modeltest 3.7 [54]. Relative rate tests [77] were performed in MEGA4 [78] using *A. longisetosus *as outgroup. Phylogenetic and genetic distance analyses were performed in PAUP* [79]. We used the branch-and-bound option to ensure finding the best tree within maximum parsimony (MP) and maximum likelihood (ML) analyses.

## Competing interests

The authors declare that they have no competing interests.

## Authors' contributions

GR provided the initial idea for the study; GR, MH, GW and ML designed the study; GR performed chemical analyses; ML performed molecular data acquisition; GW performed morphometrical analyses; MH performed molecular data analyses, combined all results and drafted the manuscript. All authors read, discussed and approved the final manuscript.
